# Productivity and Welfare Effects of Nigeria's e-Voucher-Based Input Subsidy Program

**DOI:** 10.1016/j.worlddev.2017.04.021

**Published:** 2017-09

**Authors:** Tesfamicheal Wossen, Tahirou Abdoulaye, Arega Alene, Shiferaw Feleke, Jacob Ricker-Gilbert, Victor Manyong, Bola Amoke Awotide

**Affiliations:** a International Institute of Tropical Agriculture (IITA), Abuja, Nigeria; b International Institute of Tropical Agriculture (IITA), Ibadan, Nigeria; c International Institute of Tropical Agriculture (IITA), Lilongwe, Malawi; d International Institute of Tropical Agriculture (IITA), Dar es Salaam, Tanzania; e Department of Agricultural Economics, Purdue University, 403 W. State Street, West Lafayette, USA

**Keywords:** agricultural input subsidies, welfare, productivity, smallholders, Nigeria, mobile phone

## Abstract

In an attempt to go beyond the so called “*smart*” subsidies, Nigeria has embarked on a potentially innovative mobile phone-based input subsidy program that provides fertilizer and improved seed subsidies through electronic vouchers. In this article, we examined the productivity and welfare effects of the program using household-level data from rural Nigeria. The article employed instrumental variable regression approach to control for the potential endogeneity of the input subsidy program. Our results suggest that the program is effective in improving productivity and welfare outcomes of beneficiary smallholders. The size of the estimated effects suggests a large improvement in productivity and welfare outcomes. Moreover, the distributional effects of the program suggest no heterogeneity effects based on gender and farm land size. These results are robust to using alternative measurements of program participation. The benefit–cost ratio of 1.11 suggests that the program is marginally cost-effective. Overall, our results suggest that while improving average productivity is a good outcome for improving food security, improving the distributional outcome of the program by targeting the most disadvantaged groups would maximize the program’s contribution to food security and poverty reduction.

## Highlights

• The productivity and welfare effects of an e-voucher subsidy program are evaluated.
• Instrumental variable regression employed to control for endogeneity.
• The program is effective in improving productivity and welfare outcomes.
• No heterogeneity effects based on gender and farm land size.
• The program has a modest benefit–cost ratio.

## 1 Introduction

It is widely recognized that modern agricultural technologies are critical for improving smallholder agricultural productivity. In an effort to promote adoption of yield-increasing technologies such as inorganic fertilizer and improved seed, many sub-Saharan African (SSA) countries implemented universal large-scale input subsidy programs throughout the 1970s and 1980s (Jayne et al., 2013, Jayne and Rashid, 2013, Mason and Ricker-Gilbert, 2013). However, with the introduction of the Structural Adjustment Program (SAP) in the 1980s and 1990s, such universal subsidies were greatly reduced across the region. In particular, as part of the SAP, the World Bank (WB) advised countries in SSA to phase out input subsidies on the premise that the private sector can provide it more efficiently through market-based mechanisms (Ricker-Gilbert, 2014, Ricker-Gilbert et al., 2011). However, in the late 1990s and early 2000s, large-scale targeted input subsidies have been re-introduced as a replacement of the old universal input subsidy programs (Jayne and Rashid, 2013, Jayne et al., 2013, Liverpool-Tasie and Takeshima, 2013, Lunduka et al., 2012, Ricker-Gilbert et al., 2013).^1^

Like many SSA governments, Nigeria has since then implemented a large-scale targeted input subsidy program called the Growth Enhancement Support Scheme (GES) in 2012. The program was implemented with the broad official objective of promoting agricultural productivity and food security by making fertilizer and improved seed more affordable and accessible to smallholders (Liverpool-Tasie, 2013, Liverpool-Tasie and Takeshima, 2013). The GES targets only fulltime and non-commercial farmers. In addition, it involves private agro-dealers in the procurement and distribution of subsidized fertilizer and improved seeds. In an attempt to go beyond the so called “smart” subsidies, the GES attempts to provide a 50% subsidy on two 50-kg bags of fertilizer (NPK and urea) and a 90% subsidy on a 50-kg bag of improved seeds (mostly maize and rice seeds) through e-vouchers.^2^ The e-voucher that the farmers receive via their mobile phone entitles them to buy fertilizer and improved seed from local agro-dealers at a subsidized price. The e-voucher further specifies the total quantity of fertilizer and improved seed allocated to the farmer as well as the designated redemption center for collection.

Since the GES program outsources the subsidy to private agro-dealers, it is safe to assume that the scheme will have a crowding-in effect on the supply side of fertilizer and improved seed markets in terms of private sector sales. However, farmers could still use subsidized inputs provided through the GES in place of some or all of their commercial purchases, leading to crowding out of commercial inputs on the demand side of the market. Regardless, there is little empirical evidence on the impact of the program to inform on-going debates on how effectively the GES program increases agricultural productivity and welfare of poor and food insecure households. In this study, we empirically test whether a mobile-based e-voucher system for fertilizer and improved seed subsidies in Nigeria improves productivity and welfare outcomes. Maize yield and income from maize production are used as a proxy for productivity outcomes in this article, while per-capita food, total, and non-food expenditure are used as indicators for welfare outcomes. We focus on productivity and welfare outcomes as they are the most important indicators given the stated objectives of the GES.

This article contributes to the literature on input subsidies in the following ways: First, by focusing on a country that implemented the most expensive input subsidy program in SSA, it investigates the enduring question of whether and to what extent a smart input subsidy program such as the GES impacts productivity and welfare outcomes. To date, there is not a single study that evaluated the impact of the GES program on productivity and welfare of smallholders in Nigeria. In addition, this article is the first to evaluate the effectiveness of an e-voucher input subsidy program. Second, in an attempt to provide beyond average treatment effects, we examine the distributional impacts of the GES program focusing on two sources of heterogeneity: gender and farm land size. In principle, the GES should, *ceteris paribus,* only improve the income of smallholders. However, leakages and imperfect targeting may divert subsidies away from the intended beneficiaries. Under such circumstances, the GES may become beneficial on average by improving the productivity of commercial farmers albeit ineffective in addressing the needs of smallholders. This paper therefore addresses this issue by estimating the overall average effect of the program as well as its distributional effects. In estimating the overall average and distributional impacts of the GES program, we control for the potential endogeneity of participation in the GES using Instrumental Variable (IV) regression approach. We also examined the robustness of estimated impacts by constructing alternative measures of participation in the GES program. Finally, in an attempt to provide an overall effectiveness of the program, the paper undertakes a benefit–cost analysis based on observed direct costs and benefits. The rest of the paper is organized as follows: Section 2 provides an overview of the GES program**.** Data sources and the econometric strategy are presented in Section 3. Section 4 reports the findings and discusses the results. Section 5 concludes and provides a list of open questions and discusses areas of further research.

## 2 Overview of the growth Enhancement support scheme (GES) in Nigeria

Although the vast majority of Nigeria’s rural population engages in agriculture, the sector has been in the periphery of government priorities over the past 30 years. As a result, the rate of rural poverty and food insecurity has increased substantially over that time period. For instance, rural poverty measured at the food poverty line has increased from 33.6% in 2004 to 48.3% in 2010 (National Bureau of Statistics Nigeria (NBSN), 2010). Moreover, the country’s status has changed from a food exporter to one of the world’s largest food importers, spending more than $11 billion annually (Adesina, 2012). In a stated effort to reduce this trend, the Nigerian government has embarked on the GES program in 2012. Increasing the use of improved seeds and fertilizer through “smart” subsidy schemes was seen as an essential intervention area. This policy decision was perhaps surprising because fertilizer subsidies have been in place in Nigeria in one form or another since the 1970s (Liverpool-Tasie, 2013, Liverpool-Tasie and Takeshima, 2013) and have accounted for about 40% of the total agricultural budget of Nigeria (Takeshima, Nkonya, & Deb, 2013).^3^ However, the old fertilizer subsidy program was deemed to be inefficient with widespread corruption and smuggling to neighboring countries (Olomola, 2015). Moreover, it was heavily criticized as procurement and distribution of subsidized fertilizer were mainly managed by the government with limited private sector involvement (Liverpool-Tasie & Takeshima, 2013).

The GES has been implemented with the intention to improve the efficiency of fertilizer and improved seed distribution to smallholders. By deregulating seed and fertilizer markets as well as through well-targeted fertilizer and improved seed subsidies, the GES aims to increase the productivity of smallholders. It was specifically designed to minimize leakages and to ensure that subsidies are provided only to intended farmers. In addition, the procurement and distribution of fertilizer and improved seeds is outsourced to private agro-dealers. The GES intends to provide improved seed and fertilizer to 20 million farmers within five years (Federal Ministry of Agricultural & Rural Development (FMARD), 2011). In addition, the scheme aims at increasing fertilizer use from the current level of approximately 13 kg/ha to 50 kg/ha (FMARD, 2011, Olomola, 2015).

In principle, the GES targets only fulltime and non-commercial farmers. However, there is no database of farmers to verify eligibility and most of the demographic information provided by farmers was self-reported. Registration of eligible farmers was carried out manually, with completion of forms at village level with the help of village leaders (GrowAfrica (GA), 2014, Olomola, 2015). In order to make the subsidy “*smarter*”, the initial list compiled at the village level was transferred to an electronic database. Moreover, each farmer was given a unique identifier with the help of National Identity Management Commission (NIMC). After registration was completed, eligible farmers received an e-wallet (an electronic voucher) notification through their phone. The e-wallet specifies the total quantity of fertilizer and improved seed allocated to the farmer as well as the designated redemption center for collection. According to FMARD (2011), the national electronic database of farmers registered to the GES was used to send out mobile alerts. While all registered farmers were supposed to receive a mobile alert, results from our survey suggests that some farmers have not received mobile alerts despite being registered for the GES.

## 3 Data sources and empirical strategy

### (a) Data sources

This study uses a household survey data collected by the International Institute of Tropical Agriculture (IITA) in 2015 as part of an on-going effort to evaluate the impact of adoption of drought tolerant maize varieties and the GES on productivity and welfare outcomes. A multi-stage-stratified sampling procedure was employed to select Enumeration Areas (EAs) from each Local Government Areas (LGAs), and households from each of the selected EAs. The list of all EAs was obtained from the National Population Commission (NPC) of Nigeria. The EAs were then divided by the number of LGAs in each of the selected states to obtain the number of EAs per LGA. Following the National Bureau of Statistics (NBS) recommendation for a nationally representative data collection, 10% of the LGAs in each of the selected States and 5% of the total EAs per LGA were randomly selected. Finally, from the households in each of the selected EAs, five farming households were randomly selected, resulting in a sample size of 2,305 households.

The data were collected using structured questionnaire which was pre-tested twice by trained and experienced enumerators. The survey questionnaire was designed to gather detailed information on socio-economic characteristics of households, expenditure on food, and non-food items, input use and allocation, crop and livestock production, output for maize, and other notable crops and participation in the GES program. In addition, extensive village-level data were collected on the incidence of shocks, prices of key inputs and crops, among others. In terms of participation in the GES, relevant data were collected on the level of awareness about the GES program as well as on farmers' decision to register for the GES program. In addition, data were collected on the quantity of seed and fertilizer allocated to registered farmers as well as the final quantity collected and the price paid by the participants of the GES program. To minimize errors usually encountered with the use of paper questionnaire, the data for this study were collected electronically using the “***surveybe***” software.

### (b) Empirical strategy

Like any evaluation program, establishing the causal impacts of an input subsidy program on various outcomes of interest is in fact a “wicked problem” (Ricker-Gilbert, Jayne *et al.*, 2013). First, subsidies are rarely distributed randomly across villages and among farmers. As such, identifying the causal effects of an input subsidy program requires controlling for selection bias/endogeneity stemming from observable and unobservable factors. In non-experimental data, common approaches for identifying causal impacts include different matching techniques, fixed effects (when panel data is available), and instrumental variable (IV) regression. In this paper, we employed an inverse probability-weighted adjusted regression (IPWRA) and IV regression approach due to the cross-sectional nature of our data.

One challenge with using propensity score matching is that the estimates produce biased results in the presence of misspecification in the propensity score model (Robins et al., 2007, Wooldridge, 2007, Wooldridge, 2010). For this reason, we used the IPWRA estimator which combines regression and propensity score methods in order to achieve some robustness to misspecification of the parametric models (Imbens and Wooldridge, 2009, Robins and Rotnitzky, 1995, Wooldridge, 2010). In particular, IPWRA model estimates the outcome and treatment models as follows: Suppose that the outcome model is represented by a liner regression function of the form $Y_{i}=\alpha_{i}+\theta_{i}x_{i}+\varepsilon_{i}$ for i=[01] where $Y_{i}$ is the outcome variable of interest; $x_{i}$ a set of controls; α and θ are parameters to be estimated; ε is the error term. Further, suppose that the propensity scores are given by p(x;ϑ). In the first step, we estimate the propensity scores as px;ϑ^. In the second step, we employ linear regression to estimate $\left(\alpha_{0}\text{,}\theta_{0}\right)$ and $\left(\alpha_{1}\text{,}\theta_{1}\right)$ using inverse probability-weighted least squares as follows:

(1) minα0,θ0∑iN(Yi-α0-θ0xi)/px,ϑ^ifIi=0

(2) minα1,θ1∑iN(Yi-α1-θ1xi)/px,ϑ^ifIi=1

The average treatment effect (ATT) is then computed as the difference between Eqns. (1), (2).

(3) ATT=1Nw∑iNw[α^1-α^0-(θ^1-θ^0)xi]

where (α^1) are estimated inverse probability-weighted parameters for households that participated in the GES while (α^0) are estimated inverse probability-weighted parameters for non-participants. Finally, ${\text{N}}_{\text{w}}$ stands for the total number of GES participants. $I_{i}$ is an indicator which takes a value of one if the household participates in the GES program and zero otherwise.

However, casual identification requires controlling for both observable and unobservable factors that influence participation in the GES and productivity and welfare outcomes. Hence, estimates of Eq. (3) may yield biased estimates due to biases stemming from unobservable factors. Therefore, we employed an IV regression approach to control for the potential endogeneity of participation in the GES. As mentioned above, there are several reasons for participation in the GES to be endogenous. First, households that are either more or less productive than the average smallholder may choose to register for GES. Hence, it is likely that participation in the GES is correlated with poverty status, household income, or underlying features that influence these outcome variables (Chibwana et al., 2012, Ricker-Gilbert et al., 2013, Ricker-Gilbert et al., 2011, Shively et al., 2012). Second, there is a possibility that farmers who participated in the GES share common intrinsic characteristics, such as poor/better farming skills and management abilities, which are likely to be related to poverty status and household income. As a result, we employed an IV regression approach.

However, finding an instrument that satisfies the orthogonality condition is not a trivial matter. Most of the studies that evaluated the impacts of input subsidies on such outcome indicators have used the variable “number of years the household head has lived in a village” as an instrument (Chibwana et al., 2012, Ricker-Gilbert et al., 2011, Shively et al., 2012). Following the literature, we used the number of years the household head has lived in a village as a potential instrument for participation in the GES. The number of years that the household head has lived in the village is a measure of socio-political capital that could influence farmer’s participation in the GES (Ricker-Gilbert *et al.*, 2011). We assume that this variable has no direct effect on productivity and welfare outcomes except through its effect on farmers’ decisions to participate in the GES.^4^

As explained in Section 2, participation in the GES involves a sequence of steps and choices. In our setup this process involves: becoming aware of the GES program, registering for the GES program, receiving a mobile alert and collecting subsidized fertilizer and improved seeds from redemption centers. Registration is a strict subset of awareness, and receiving a mobile alert is a strict subset of registration in the GES. However, collection of subsidized fertilizer and improved seeds is not a strict subset of receiving a mobile alert (it is a strict subset of registration). In order to capture the above sequential process, we employed the following sequential probit model.

(4) PrIk,i=1|Xi,Si,Vi,Zi,Ik-1,i=1=p^ik

where p^ik is the standard probit model represented by: p^ik=Φ(Xi,Si,Vi,Zi,β). Herein, Φ represents the cumulative distribution function and β captures vectors of parameters to be estimated. $I_{k}$ is an indicator function that takes a value of one if farmer i passes transition k (becoming aware of the GES, registering for the GES, and receiving a mobile alert) and zero otherwise. $X_{i}$ captures a vector of household *i*’s characteristics such as household size, age, education that affect participation in the GES. *S_i_* captures participation in social network activities such as membership in cooperatives and labor-sharing arrangements (Wossen, Berger, & Di Falco, 2015) while $V_{i}$ captures state-level fixed effects to control state-level heterogeneity in the implementation of the GES program. The variable $Z_{i}$ is our instrument: the number of years the household head has resided in the village. According to FMARD (2011), all registered farmers will receive mobile alerts. As such, the selection bias occurs at the awareness and registration stage. We assume that those farmers who have lived in the village for long time are more likely to be aware of the GES program as they have more connections. Similarly, they are also more likely to be recognized by village leaders and hence can be considered as genuine farmers at the time of registration, which increases the likelihood of participating in the GES.

The outcome equation estimates the effect of participation in the GES program on productivity and welfare indicators. Formally, the empirical specification is presented as follows:

(5) $$Y_{\mathrm{ij}}=\alpha_{0}+\tau I_{\mathrm{ij}}+\beta X_{\mathrm{ij}}+\gamma S_{\mathrm{ij}}+\vartheta V_{\mathrm{ij}}+\varepsilon_{\mathrm{ij}}\text{.}$$

In the above equation, the predicted probability of participation from the first stage probit model is used as instrument for $I_{\mathrm{ij}}$ (participation in the GES). This method is efficient even with weak instruments and it is preferred to other IV methods since our treatment variable is binary (Wooldridge, 2007).

### (c) Outcome indicators

The outcome indicators are related to productivity and welfare. Our first productivity outcome-related indicator is maize yield. Although the GES does not require participating farmers to use the subsidized inputs on a particular crop, we opt to consider maize yield as a productivity outcome variable as maize is one of the most important food crops in Nigeria. In fact, Nigeria is the largest maize producer in West Africa. In addition, maize also stands to benefit the most from fertilizer subsidies as response rate of improved maize is higher than most other crops. According to our data, average maize yield in the study area stands at 2,006 kg/ha. However, average maize yield for GES participants (2,205 kg/ha) is significantly higher than for non-participants (1,860 kg/ha) and the difference is statistically significant at 1% significance level (see Table 1). Our second productivity-related indicator is measured by income from maize production.^5^ Looking into the distribution of income from maize production, income received from maize sales is higher for GES participants (Table 1). However, these differences in maize yield and income cannot simply be attributed to the GES by looking at the mean differences between GES participants and non-participants. In particular, these mean differences are only indicative of correlations and cannot be used to make causal inferences regarding the impacts of the GES on maize yields and income without controlling for other confounding factors.

Our welfare-related indicators include food expenditure, non-food expenditure, and total expenditure, all measured on per-capita basis. In addition to expenditure indicators, we also used headcount poverty ratio as an additional welfare indicator. Total expenditure is calculated by summing food and non-food expenditure values. A household’s food consumption expenditure is comprised of monetary expenditures on purchased food and the imputed values of consumption from own harvest. Looking into the distribution of consumption expenditures, the average per capita total consumption expenditure is about ₦112,136 per year.^6^ Like productivity indicators, we found significant differences in per-capita food, non-food, and total consumption expenditures between participants and non-participants of the GES (see Table 1). However, as mentioned above, these differences between participants and non-participants cannot be attributed to the GES. Our final welfare related outcome indicator measures the proportion of households below the poverty line, commonly referred as the headcount ratio. Following Foster, Greer, and Thorbecke (1984), per-capita total expenditure is used to determine households’ poverty status. Formally, headcount ratio ($P_{0}$) is calculated as:

(6) $$P_{0}=\frac{1}{N}\overset{N}{\underset{i=1}{\sum }}I(X_{p}<z)\text{.}$$

where $X_{p}$ is per-capita total expenditure and N is the relevant population size. z is a scalar set at per capita total expenditure level of ₦91,250 per year per capita (NBSN, 2010).^7^ I(.) is an indicator function which takes on a value of 1 when $X_{p}<z$ and a value of zero when $X_{p}\geq z$.

### (b) Descriptive statistics

In the household survey, we collected detailed information regarding the awareness and registration process of the GES. More specifically, first we asked households if they were aware of the GES program. Second, we asked those who responded in the affirmative if they had registered in the GES program. Third, we asked those who responded to the registration question in the affirmative if they had received mobile alert. Finally, we asked those who were notified of the e-voucher through the mobile alert if they had actually collected the subsidized inputs and the quantity of seed and fertilizer allocated to them as well as the final quantity they collected. We used the above sequence of questions to construct dummy variables for our sequential probit model. According to the survey, about 65.5% of the households were aware of the GES program and only 64% of them registered for the GES. However, our survey further revealed that only 76% of the registered households actually received mobile alerts.

Since our main objective is to evaluate the overall effectiveness of the GES program on productivity and welfare outcomes, we used registration to the GES program as our main treatment variable. In particular, participation in the GES is measured by a dummy variable which takes a value of one if the household is registered to the GES and zero otherwise. Note that, in the robustness section, we used receiving a mobile alert as well as actual collection of subsidized fertilizer and improved seed as our treatment variables. Table 1 presents the descriptive statistics of the key variables of interest based on the registration status of households. We included household characteristics such as age, household size, education, membership in different social groups, risk-aversion, self-reported weather, and stress shocks as well as wealth indicators such as TLU and land size. In addition, we have included access to climate and improved seed information as these variables affect awareness and registration decision to the GES. We assume that the above key household characteristics affect farmers' ability to participate in the GES. For instance, we hypothesize that the education level of the household head affects the likelihood of participation in the GES positively. However, for most of our controls, the direction of expected impacts cannot be determined a priori. The variable risk-aversion is measured by farmer's willingness to try new agricultural practices such as improved seed. In particular, data were collected on how willing the farmers are to take risks related to new improved maize varieties. We consider farmers as risk-averse if they are unwilling to ever try new improved varieties. However, given the proxy nature of our measurement, its effect should be interpreted with caution.

In addition to household characteristics, we also included state dummies to control for state-level fixed effects. We further have controls for general conditions such as access to electricity. Finally, the number of years of residence in the village serves as an instrument for participation in the GES. Table 1 further presents the difference in means between participant and non-participant based on registration to the GES. As shown in Table 1, the average household size is about 7.5 members for the whole sample. While comparing household size between GES participants (7.9 members) and non-participants (7.3 members), we found significant difference between the two groups. Moreover, most of the respondents are literate, the average literacy rate being 84%. The average age of the household head is 47 years. About 90% of the household heads are male and married. In our sample about 30% have access to off-farm employment opportunities. In terms of self-reported shocks, about 67% and 19% of the respondents have experienced stress and drought shocks, respectively. On average, GES participants have better access to off-farm employment, climate and varietal information, and housing condition and tend to be older. Moreover, GES participants reported higher incidence of stress and drought shock compared to non-participants. We also found significant difference between the two groups in terms of the number of years the household head has resided in the village.

**Table 1:** Descriptive statistics by GES registration

	Full sample^a^ (*N* = 1,919)	Registered to the GES (*N* = 812)	Not registered to the GES (*N* = 1,107)	Mean diff
Maize yield (kg/ha)	2,006	2,205	1,860	345^***^
Income from maize production (₦)	77,517	88,216	69,669	45,935^***^
Per capita total expenditure (₦)	112,136	145,213	87,874	57,339^***^
Per capita food expenditure (₦)	53,890	66,310	44,779	21,531^***^
Per capita non-food expenditure (₦)	58,246	78,092	43,095	35,807^***^
Household size	7.55	7.9	7.3	0.6^***^
Mobile phone ownership	0.93	0.94	0.92	0.02
Education (1 = literate, 0 = otherwise)	0.84	0.84	0.838	−0.002
Marital status (1 = married, 0 = otherwise)	0.90	0.91	0.89	0.01
Age of the household head	47.3	49.1	45.97	3.1^***^
Gender of the household head (1 = Female, 0 = otherwise)	0.105	0.096	0.111	−0.015
Access to off-farm work (1 = has access, 0 = otherwise)	0.30	0.36	0.25	0.11^***^
Roofing material of the house (1 = has a sheet)	0.88	0.90	0.86	0.04^***^
Farm size (ha)	4.47	4.64	4.34	0.30^*^
Stress shock (1 = experience stress shock, 0 = otherwise)	0.66	0.73	0.62	0.11^***^
Drought shock(1 = experience drought, 0 = otherwise)	0.19	0.24	0.15	0.09^***^
Membership to credit and saving groups (1 = yes, 0 = No)	0.078	0.094	0.067	0.027^**^
Membership to labor-sharing groups (1 = yes, 0 = No)	0.036	0.045	0.029	0.016^*^
Membership to cooperatives (1 = yes, 0 = otherwise)	0.10	0.15	0.07	0.08^***^
Membership to farmer research group (1 = yes, 0 = No)	0.15	0.225	0.103	0.12^***^
Access to varietal information (1 = yes, 0 = No)	0.17	0.23	0.12	0.11^***^
Access to climate information (1 = yes, 0 = No)	0.58	0.70	0.52	0.18^***^
Risk aversion (1 = willing to try new things, 0 = otherwise)	0.74	0.82	0.68	0.12^***^
Number of years residence in the village (years)	38.8	48.3	31.9	16.4^***^
Access to electricity (1 = yes, 0 = otherwise)	0.48	0.53	0.44	0.9^***^

a Our final sample includes 1,919 households due to missing values for expenditure, yield, and other controls.

## 4 Empirical results

In this section, we present the results of our econometric analysis. Firstly, we report the effect of the GES program on productivity, maize income, and welfare outcomes using matching techniques in Table 2. We then proceed to present effects estimated using the IV regression approach in Table 5 for productivity and maize income and in Table 6 for welfare indicators. In addition to average treatment effects, in Table 5, Table 6 we present distributional effects of the program estimated based on interaction effects. We present results for alternative measure of participation in the GES program in Table 7, Table 8 and placebo effects in Table 9. Finally, in Table 10, we present benefit–cost ratios.

### (a) Matching results

Table 2 presents PSM and IPWRA estimation results for the following outcome indicators: (i) maize yield; (ii) income from maize production; (iii) per-capita food expenditure; (iv) per-capita non-food expenditure; (v) per-capita total expenditure; and (vi) poverty headcount ratio.^8^ We base our interpretation based on IPWRA results as they are more robust than PSM. We find a positive and statistically significant effect of participation in the GES program on all productivity and welfare outcome indicators. The results show that participation in the GES program increased maize yields and maize income by 22% and 26% respectively. In terms of welfare outcomes, we found positive and statistically significant effects on consumption and a negative and statistically significant effect on poverty headcount ratio. In particular, the probability of being poor declined by 24% points as a result of the GES program. However, these results have to be interpreted with caution and in fact they may be biased since we did not control for unobserved heterogeneity.

**Table 2:** Impacts of GES participation on outcomes of interest using PSM and IPWRA estimation

Treatment variable = 1 if household is registered for GES	PSM	IPWRA
Maize yield	0.21^***^	0.22^***^
	(0.053)	(0.046)
Income from maize production	0.32^***^	0.259^***^
	(0.071)	(0.07)
Food expenditure	0.52^***^	0.60^***^
	(0.062)	(0.040)
Total expenditure	0.401^***^	0.46^***^
	(0.061)	(0.043)
Non-food expenditure	0.48^***^	0.496^***^
	(0.08)	(0.061)
Poverty headcount ratio	−0.21^***^	−0.24^***^
	(0.031)	(0.025)
*N*	1,919	1,919

Robust standard errors in bracket, ^***^p < 0.01, ^**^p < 0.05, ^*^p < 0.1.

The reliability of the PSM and IPWRA results depends on the quality of our matching. We therefore provide some details on the overall covariate balancing and common support. Table 3 presents the overall covariate balancing test before and after matching. The results reveal that the standardized mean difference for all covariates used in the PSM is reduced from 19.6% pre-matching to 3.7% post-matching. This result shows that matching reduces bias by about 81%. In addition, we rejected the joint significance of covariates post-matching (*p*-value = 0.543) while the joint significance of covariates was not rejected before matching (*p*-value = 0.0000). Moreover, due to matching, the pseudo-*R*^2^ declined from 15.2% to 1.1%.

**Table 3:** Propensity score matching quality test.

	Pseudo *R*^2^	LR *X*^2^	*p*-Value	Mean bias
Before matching	0.152	397.28	0.0000	19.6
After matching	0.011	22.6	0.543	3.7

**Figure 1:**
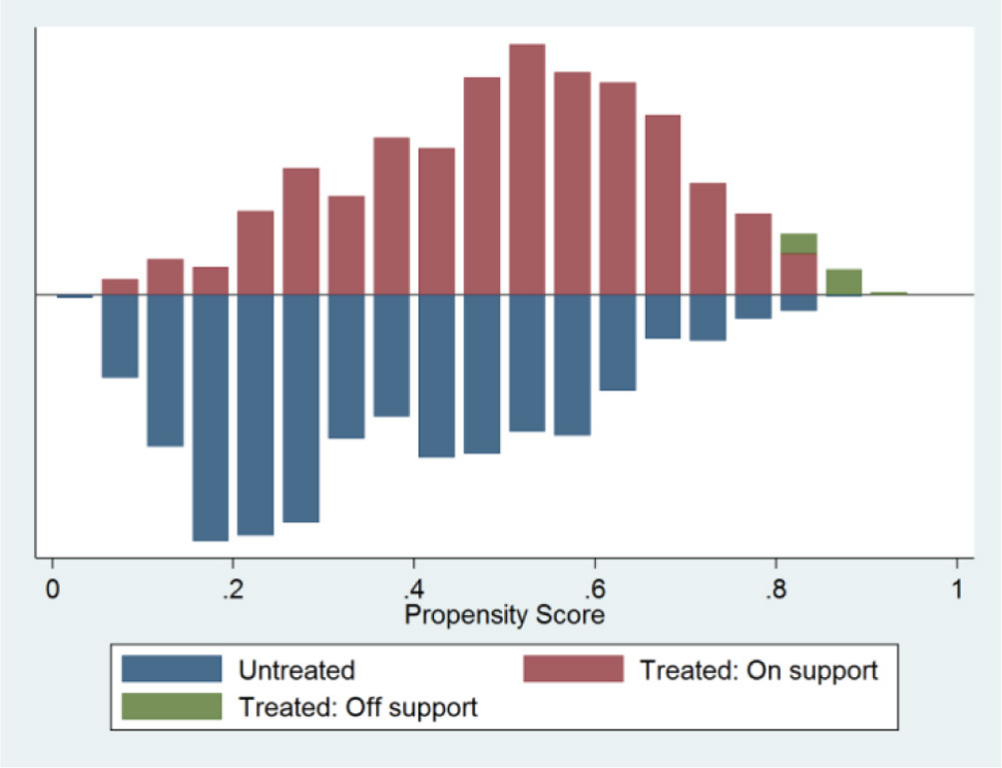
Distribution of propensity scores and common support region.

The high total bias reduction, the insignificant p-values of the likelihood ratio test after matching, low pseudo-*R*^2^, and significant reduction in the mean standardized bias are indicative of successful balancing of the distribution of covariates between participants and non-participants of the GES. The common support region is presented in Figure 1. A visual inspection of the distribution of the estimated propensity scores indicates that the common support condition is satisfied as there is substantial overlap in the distribution of the propensity scores of both participants and non-participants of the GES.

### (b) IV estimation results

#### (i) Determinants of participation in the GES: Sequential probit model

As mentioned in the methodology section, we used a sequential probit model to examine the determinants of participation in the GES due to the sequential steps involved in the GES program. In our setting, first we estimate the determinants of awareness to the GES. We then estimate the determinants of registration to the GES conditional on awareness. Finally, we estimate the determinants of receiving a mobile alert conditional on registration. We followed this step as registration is a strict subset of awareness. Similarly, receiving a mobile alert is a strict subset of registration. We did not include the decision to collect allocated quantities of fertilizer and improved seed in our sequential probit model as it is not a strict subset of receiving a mobile alert.^9^ Since the selection bias occurs at the awareness and registration stage, we assume that our instrument and farmer characteristics affects awareness and registration decision but not the probability of receiving a mobile alert conditional on awareness and registration.^10^

Results of the sequential probit model presented in Table 4 indicate that the excluded IV (*Number of years of residence in the village)* affects the probability of becoming aware of the GES and the conditional probability of registration to the GES conditional on awareness but not the conditional probability of receiving a mobile alert given registration in the GES (Table 4). This shows that the selection bias has been remedied by the instrument at the level of awareness and registration. Looking into the determinants of participation at the different stages of the GES, we found that some farmer characteristics such as access to climate information, access to varietal information, social capital variables, and education affect only awareness about the GES but not the probability of registration conditional on awareness and the probability of receiving mobile alerts conditional on registration. Similarly, access to off-farm income and quality of housing, a proxy for wealth, affects awareness about the GES as well as the probability of registration conditional on awareness positively.

**Table 4:** Estimates of sequential probit model

Variable	GES awareness	GES registration	Received mobile alert
Mobile phone ownership	0.101	−0.035	−0.177
	(0.134)	(0.173)	(0.308)
Household size	0.005	0.008	−0.001
	(0.008)	(0.010)	(0.012)
Education	0.284^***^	−0.175	0.233
	(0.089)	(0.136)	(0.174)
Marital status	−0.356^**^	0.372^*^	−0.252
	(0.157)	(0.225)	(0.352)
Age of the household head	−0.026^**^	0.009	0.043
	(0.012)	(0.024)	(0.035)
Gender of the household head	−0.228	0.477^**^	−0.115
	(0.140)	(0.210)	(0.287)
Access to off-farm	0.203^***^	0.221^**^	0.019
	(0.069)	(0.093)	(0.134)
Roofing material of the house	0.283^***^	0.485^***^	−0.442
	(0.104)	(0.165)	(0.476)
Stress shock	0.083	0.144	0.176
	(0.098)	(0.100)	(0.125)
Drought shock	0.125	0.363^***^	−0.071
	(0.087)	(0.113)	(0.131)
Membership to credit and saving groups	0.176	0.134	−0.104
	(0.129)	(0.171)	(0.211)
Membership to cooperatives	0.319^**^	0.396	−0.243
	(0.149)	(0.262)	(0.171)
Membership to farmer research group	−0.040	0.021	0.153
	(0.067)	(0.108)	(0.114)
Risk aversion	0.109	0.189^*^	0.086
	(0.089)	(0.107)	(0.294)
Access to electricity	0.065	0.114	−0.038
	(0.090)	(0.086)	(0.132)
Access to varietal information	0.174^*^	0.070	0.155
	(0.092)	(0.147)	(0.171)
Access to climate information	0.491^***^	0.139	−0.135
	(0.071)	(0.089)	(0.172)
Number of years of residence in the village	0.026^***^	0.041^***^	−0.005
	(0.002)	(0.003)	(0.004)
State fixed effects	Yes	Yes	Yes
Wald chi2(27)	334.1^***^	332.5^***^	61.4^***^
Pseudo *R*^2^	0.17	0.32	0.05
Goodness of fit measure (McIntosh and Dorfman (1992)	1.34	1.54	0.87
Percentage correct predictions	0.705	0.79	0.87
*N*	1,919	1,256	615

Standard errors clustered at enumeration area level are reported in parentheses, ^***^p < 0.01, ^**^p < 0.05, ^*^p < 0.1.

This result is consistent with the findings of Ricker-Gilbert *et al.* (2011) and Chibwana *et al.* (2012) in Malawi. In addition, neither farmer characteristics nor the number of years the household head has resided in the village affect the probability of receiving mobile alerts conditional on registration to the GES. We added goodness of fit measure such as (Wald chi^2^, Pseudo *R*^2^, percentage of correct prediction, and goodness of fit measure based on McIntosh and Dorfman (1992)) at the bottom of Table 4. All the goodness of fit measures suggest a very high fit for our probit model.

#### (ii) Effects on productivity and maize income

The result showing the effects of the GES program on maize yields and maize income is presented in Table 5. Estimates of “*No control*” present a parsimonious specification for maize yield and income that includes only the treatment variable (registration for the GES) along with state-level fixed effects. Estimates of “*With control*”, present results where standard controls for maize yield and income are included. Estimates of “*With IV*” present the results of the IV specification in which participation in the GES program is treated as endogenous. We specify a log-linear functional form for maize yield and income from maize production is estimated at levels.

The results presented in Table 5 show that participation in the GES program has a positive and statistically significant effect on maize yields and income. Looking into our parsimonious model specification for maize yield, we found that the effect of the GES is similar in terms of the magnitude and direction of estimated impacts in all specifications. In particular, farmers who participated in the GES increased their maize yields by 28.1% when we controlled only for state-level fixed effects, 26.1% when we included standard controls in addition to state-level fixed effects and by 26.3% when we controlled for the potential endogeneity of participation in the GES. Similarly, estimated results in Table 5 suggest an increase of maize income by ₦1.59 to ₦2.53. Note that, values for maize income are expressed in ₦10,000. For instance, the effect size ₦1.59 implies that GES participation increases income from maize production by ₦15,900.

In our subsequent discussion, we base all our interpretations based on our preferred specification (IV specifications). The results in Table 5 show that as a result of GES, maize yield has increased by 26.3%. Similarly, maize income of GES participants has increased by ₦19,730. These results suggest that the GES program enabled farmers to improve their productivity and maize income. In Table 5, we also included interaction terms to test if participation in the GES has heterogeneous impacts. In particular, we introduced an interaction term between participation in the GES and the gender of the household head as well as between participation in the GES and farm land size. We created a dummy variable called “*land category*” which takes on a value of one if a household owns less than 3 hectares of farm land and zero otherwise.^11^ The results show that the interaction term between the GES and the gender of the household head is insignificant for both maize yield and maize income. These results suggest that GES benefitted female-headed households (FHHs) as much as male-headed households (MHHs). Similarly, the interaction term between the GES and land category is insignificant.

**Table 5:** Effect of the GES on maize yield and maize income

	Maize yield			Maize income		
	No control	With control	With IV	No control	With control	With IV
GES	0.281^***^	0.261^***^	0.263^**^	1.59^***^	2.530^***^	1.973^*^
	(0.04)	(0.060)	(0.118)	(0.41)	(0.838)	(1.161)
Gender		−0.221^*^	−0.346^**^		0.617	1.969^*^
		(0.122)	(0.147)		(1.429)	(1.10)
Land category		0.035	0.043		−0.651	-3.060^***^
		(0.073)	(0.094)		(0.820)	(0.701)
GES^*^Gender		0.025	0.372		−0.883	-3.566
		(0.120)	(0.254)		(2.085)	(2.347)
GES * land category		−0.081	−0.103		-1.117	−0.802
		(0.082)	(0.139)		(1.293)	(1.234)
Household size		−0.003	−0.003		1.293^***^	0.137^***^
		(0.007)	(0.007)		(0.420)	(0.043)
Education		0.035	0.030		0.620	1.437^***^
		(0.050)	(0.051)		(0.564)	(0.433)
Marital status		−0.042	−0.021		0.158	1.781^**^
		(0.106)	(0.110)		(0.257)	(0.750)
Age		0.014	0.012		1.988^***^	−0.019
		(0.016)	(0.016)		(0.461)	(0.061)
Age squared		−0.000	−0.000		−0.013	−0.000
		(0.000)	(0.000)		(0.045)	(0.001)
Access to off-farm		0.005	0.005		1.833	−0.504
		(0.047)	(0.047)		(1.171)	(0.376)
Roofing material of the house		0.210^***^	0.208^***^		1.952	−0.063
		(0.066)	(0.067)		(1.281)	(0.746)
Stress shock		0.118^**^	0.116^**^		−0.001	1.928^***^
		(0.055)	(0.057)		(0.001)	(0.520)
Member to credit and saving groups		0.044	0.044		−0.445	0.090
		(0.061)	(0.061)		(0.835)	(0.779)
Member to labor-sharing groups		−0.167^*^	−0.168^*^		0.267	1.275
		(0.089)	(0.089)		(0.961)	(1.378)
Member to cooperatives		0.198^**^	0.187^**^		1.855^**^	−0.229
		(0.077)	(0.077)		(0.869)	(0.937)
Member to farmer research group		−0.046	−0.045		-1.183	0.304
		(0.042)	(0.042)		(0.720)	(0.411)
Risk aversion		−0.023	−0.022		0.577	1.329^**^
		(0.053)	(0.053)		(1.254)	(0.565)
Access to electricity		−0.010	−0.012		6.023^***^	0.177
		(0.043)	(0.044)		(1.782)	(0.394)
Access to varietal information		0.035	0.031		-1.198	1.339^**^
		(0.063)	(0.061)		(1.271)	(0.556)
Access to climate information		0.013	0.011		0.750	0.703
		(0.052)	(0.051)		(0.597)	(0.471)
Joint significance of all regressors (F-test)	15.9^***^	5.7^***^	4.86^***^	7.21^***^	9.68^***^	8.85^***^
*R*^2^	0.055	0.08	0.07	0.03	0.24	0.11
Observations	1,919	1,919	1,919	1,919	1,919	1,919

State fixed effects but not reported here. Standard errors clustered at enumeration area level are reported in parentheses, ^***^p < 0.01, ^**^p < 0.05, ^*^p < 0.1. *Note*: maize yield is estimated in log.

#### (iii) Effects on welfare outcome indicators

This section presents and discusses the welfare effects of the GES program. We used per-capita total expenditure, food expenditure as well as poverty headcount ratio as indicator for welfare. In our estimation, total expenditure and food expenditure are measured on per capita basis and estimated using a log-linear functional form. Results are reported in Table 6 Results.^12^ Like the previous section, we did parsimonious specifications in which we estimated effects without standard controls, with standard controls, and with IV. Our discussion is based on IV results. Our main result shows that the GES program has a positive and statistically significant effect on per-capita total expenditure and food expenditure. In addition to the direction of the estimated impacts, the effect size suggests a large improvement in welfare outcomes as a result of participation in the GES program.

On average, per-capita total expenditure increased by 30.7%. Similarly, as a result of participation in the GES program, per-capita food consumption expenditure increased by 39.4%. These results are consistent with previous studies in Nigeria. For instance, Awotide, Karimov, Diagne, and Nakelse (2013) found that certified improved rice seed voucher system, which entitles beneficiaries to up to 20 kg of seed at subsidized price, increased annual household income and per capita consumption expenditure per annum and subsequently contributed to overall poverty reduction by about 24% points. Similar to our previous specification, we also included an interaction term between the GES and gender as well as farm land size and found insignificant effects for both per-capita total expenditure and food expenditure.

The final welfare indicator, headcount poverty ratio, is a binary variable and hence the effect size can be estimated using a probit model specification. However, the parameter estimates for GES will only represent changes in the probability of poverty instead of actual poverty reduction rates. Estimating the effect of GES on poverty reduction instead of changes in the probability of poverty reduction requires examining the distribution of observed poverty of GES participants and the distribution of the counterfactual poverty of GES participants had they not participated in the GES program. In other words, we need to examine the poverty reduction effects of the 30.7% per-capita total expenditure growth reported in Table 6 above and examine whether such changes are of sufficient magnitude to lift poor farmers above the poverty line. We therefore, calculated changes in the headcount poverty ratio of GES participants as a result of the 30.7% increase in per-capita consumption expenditure. Our result suggests that as a result of GES, headcount poverty ratio has declined by 17.7% points.

**Table 6:** Effect of the GES on food and total consumption expenditure

	Per-capita total expenditure			Per-capita food expenditure		
	No control	With controls	With IV	No control	With controls	With IV
GES	0.51^***^	0.396^***^	0.307^**^	0.64^***^	0.556^***^	0.394^**^
	(0.046)	(0.081)	(0.143)	(0.05)	(0.091)	(0.191)
Gender		−0.246^**^	−0.328^**^		−0.224^*^	−0.363^**^
		(0.122)	(0.161)		(0.126)	(0.168)
Land category		−0.097	−0.168		−0.063	−0.159
		(0.078)	(0.114)		(0.079)	(0.124)
GES^*^Gender		0.011	0.252		0.052	0.451
		(0.140)	(0.288)		(0.141)	(0.299)
GES^*^land category		0.118	0.266		0.058	0.261
		(0.129)	(0.214)		(0.133)	(0.234)
Education		0.162^**^	0.161^**^		0.066	0.063
		(0.066)	(0.067)		(0.065)	(0.066)
Marital status		−0.307^*^	−0.293^*^		−0.185	−0.160
		(0.157)	(0.159)		(0.157)	(0.164)
Age		−0.004	−0.005		−0.011	−0.012
		(0.014)	(0.014)		(0.013)	(0.015)
Age squared		−0.000	0.000		0.000	0.000
		(0.000)	(0.000)		(0.000)	(0.000)
Access to off-farm		0.069	0.075		0.015	0.025
		(0.051)	(0.051)		(0.048)	(0.048)
Roofing material of the house		−0.059	−0.053		−0.069	−0.056
		(0.084)	(0.081)		(0.077)	(0.079)
Stress shock		0.329^***^	0.330^***^		0.371^***^	0.374^***^
		(0.052)	(0.054)		(0.055)	(0.058)
Member to credit and saving groups		0.015	0.012		−0.018	−0.022
		(0.067)	(0.074)		(0.065)	(0.073)
Member to labor-sharing groups		−0.002	0.002		0.041	0.047
		(0.109)	(0.103)		(0.126)	(0.117)
Member to cooperatives		0.085	0.078		0.086	0.084
		(0.091)	(0.098)		(0.089)	(0.101)
Member to farmer research group		−0.060	−0.058		−0.039	−0.036
		(0.052)	(0.055)		(0.046)	(0.049)
Risk aversion		0.290^***^	0.297^***^		0.234^**^	0.245^**^
		(0.090)	(0.097)		(0.111)	(0.122)
Access to electricity		0.140^***^	0.140^***^		0.160^***^	0.162^***^
		(0.046)	(0.046)		(0.050)	(0.049)
Access to varietal information		0.132^**^	0.135^**^		0.096^*^	0.103^*^
		(0.058)	(0.059)		(0.055)	(0.057)
Access to climate information		0.122^*^	0.125^*^		0.090	0.099
		(0.065)	(0.071)		(0.063)	(0.073)
Joint significance of all regressors (F-test)	72.5^***^	27^***^	22.7^***^	61.52^***^	23.31^***^	16.15^***^
*R*^2^	0.21	0.27	0.24	0.19	0.24	0.19
Observations	1,919	1,919	1,919	1,919	1,919	1,919

State-level fixed effects included but not reported. Standard errors clustered at enumeration area level are reported in parentheses, ^***^p < 0.01, ^**^p < 0.05, ^*^p < 0.1. Note: Per-capita food expenditure and total expenditure are measured in per capita and transformed in logarithmic scale.

### (c) Robustness check for GES measurement

In this section we present robustness cheeks for alternative measures of participation in the GES program.

#### (i) Measuring treatment based on mobile alerts

In our main analysis, we measure participation in the GES by registration status. Our data show that only registered farmers have received mobile alerts. However, of the registered farmers, only 76% have received mobile alerts. As a robustness check, we define treatment based on receiving a mobile alert. In particular, we created a dummy variable which takes a value of one if the household has received a mobile alert and zero otherwise.

Results are reported in Table 7 above. Parameter estimates for maize yield and per-capita total expenditure are consistent with our main findings in the results section of the paper.

**Table 7:** . Results based on receiving mobile alerts

	Maize yield			Per-capita total expenditure		
	No control	With controls	With IV	No control	With controls	With IV
GES	0.237^***^	0.219^***^	0.194^**^	0.54^***^	0.413^***^	0.349^***^
	(0.04)	(0.058)	(0.085)	(0.048)	(0.071)	(0.087)
Gender		−0.241^**^	−0.324^**^		−0.262^**^	−0.318^**^
		(0.121)	(0.138)		(0.116)	(0.140)
Land category		0.028	0.022		−0.104	−0.121
		(0.064)	(0.070)		(0.067)	(0.074)
GES*Gender		0.096	0.375^*^		0.049	0.246
		(0.122)	(0.223)		(0.151)	(0.265)
GES*land category		−0.099	−0.074		0.131	0.195
		(0.079)	(0.108)		(0.122)	(0.148)
Education		0.025	0.021		0.131^**^	0.132^*^
		(0.051)	(0.051)		(0.066)	(0.067)
Marital status		−0.035	−0.020		−0.297^*^	−0.287^*^
		(0.106)	(0.108)		(0.155)	(0.155)
Age		0.015	0.013		−0.003	−0.004
		(0.015)	(0.015)		(0.013)	(0.014)
Age squared		−0.000	−0.000		−0.000	0.000
		(0.000)	(0.000)		(0.000)	(0.000)
Access to off-farm		0.017	0.017		0.089^*^	0.092^*^
		(0.046)	(0.046)		(0.049)	(0.050)
Roofing material of the house		0.223^***^	0.223^***^		−0.052	−0.044
		(0.067)	(0.067)		(0.086)	(0.082)
Stress shock		0.127^**^	0.126^**^		0.346^***^	0.346^***^
		(0.053)	(0.055)		(0.052)	(0.054)
Member to credit and saving groups		0.044	0.041		0.009	0.008
		(0.060)	(0.061)		(0.069)	(0.074)
Member to labor-sharing groups		−0.155^*^	−0.153^*^		0.013	0.018
		(0.088)	(0.088)		(0.109)	(0.105)
Member to cooperatives		0.204^***^	0.198^**^		0.091	0.084
		(0.078)	(0.077)		(0.093)	(0.094)
Member to farmer research group		−0.047	−0.044		−0.059	−0.057
		(0.042)	(0.042)		(0.051)	(0.054)
Risk aversion		−0.019	−0.016		0.291^***^	0.297^***^
		(0.053)	(0.052)		(0.088)	(0.093)
Access to electricity		−0.009	−0.012		0.133^***^	0.133^***^
		(0.044)	(0.044)		(0.045)	(0.046)
Access to varietal information		0.035	0.034		0.122^**^	0.126^**^
		(0.061)	(0.061)		(0.057)	(0.057)
Access to climate information		0.020	0.020		0.126^**^	0.128^**^
		(0.052)	(0.051)		(0.063)	(0.065)
Joint significance of all regressors (F-test)	12.66^***^	5.24^***^	4.85^***^			
81.26^***^						
26.42^***^						
23.3^***^						
R^2^	0.05	0.07	0.07	0.21	0.27	0.244
Observations	1,919	1,919	1,919	1,919	1,919	1,919

State-level fixed effects included but not reported. Standard errors clustered at enumeration area level are reported in parentheses, ^***^p < 0.01, ^**^p < 0.05, ^*^p < 0.1. *Note****:*** Per-capita total expenditure is measured in per capita and transformed in logarithmic scale.

#### (ii) Measuring treatment based on actual collection of subsidized inputs

Our second robustness check is based on actual collection of subsidized fertilizer and improved seed by farmers. Our data show that only registered farmers have managed to collect subsidized fertilizer and improved seed. However, only 69% of the registered farmers have collected subsidized fertilizer and improved seed. Similarly, 3.7% of the registered farmers have managed to collect subsidized fertilizer and improved seed without receiving a mobile alert. The second case may arise due to network coverage or mobile phone ownership issues. The program clearly stated that when the use of mobile phone is not tenable, farmers can claim benefits using scratch cards or GES identification cards. As such the second group of the beneficiaries may have collected their share of fertilizer and improved seed through scratch cards or GES identification cards. However, the first group of households may have failed to collect their share due to liquidity constraint as farmers are expected to pay the subsidized price to private agro-dealers or the redemption centers may have been far away which makes the final price expensive due to high transaction costs. Nonetheless, these two issues will introduce measurement errors. Herein, we probe the robustness of our results by measuring treatment by a dummy variable which takes on a value of one if the household has collected subsidized inputs and zero otherwise as a measure of program participation. We focus on maize yield and poverty outcomes. Results are reported in Table 8. Parameter estimates for maize yield and per-capita total expenditure are consistent with our previous findings.

**Table 8:** Results based on actual collection of subsidized inputs

	Maize yield			Per-capita total expenditure		
	No control	With control	With IV	No control	With control	With IV
GES	0.223^***^	0.235^***^	0.237^**^	0.54^***^	0.426^***^	0.424^***^
	(0.04)	(0.054)	(0.103)	(0.026)	(0.070)	(0.104)
Gender		−0.213^*^	−0.317^**^		−0.260^**^	−0.330^**^
		(0.118)	(0.141)		(0.114)	(0.142)
Land category		0.041	0.029		−0.080	−0.130^*^
		(0.060)	(0.073)		(0.061)	(0.078)
GES^*^Gender		0.000	0.411		0.077	0.356
		(0.116)	(0.273)		(0.133)	(0.325)
GES^*^land category		−0.145^*^	−0.103		0.096	0.251
		(0.078)	(0.131)		(0.111)	(0.177)
Education		0.025	−0.002		0.127^*^	0.114^*^
		(0.051)	(0.007)		(0.067)	(0.068)
Marital status		−0.037	0.016		−0.281^*^	−0.262^*^
		(0.105)	(0.052)		(0.155)	(0.158)
Age		0.015	−0.009		−0.004	−0.006
		(0.015)	(0.109)		(0.014)	(0.014)
Age squared		−0.000	0.012		0.000	0.000
		(0.000)	(0.015)		(0.000)	(0.000)
Access to off-farm		0.016	−0.000		0.090^*^	0.089^*^
		(0.046)	(0.000)		(0.049)	(0.050)
Roofing material of the house		0.233^***^	0.016		−0.033	−0.034
		(0.067)	(0.046)		(0.090)	(0.082)
Stress shock		0.125^**^	−0.078^*^		0.341^***^	0.338^***^
		(0.053)	(0.045)		(0.052)	(0.054)
Member to credit and saving groups		0.054	0.013		0.023	0.022
		(0.061)	(0.068)		(0.070)	(0.074)
Member to labor-sharing groups		−0.155^*^	0.049		0.012	0.015
		(0.087)	(0.061)		(0.105)	(0.105)
Member to cooperatives		0.223^***^	−0.156^*^		0.121	0.105
		(0.078)	(0.088)		(0.096)	(0.094)
Member to farmer research group		−0.053	0.211^***^		−0.072	−0.072
		(0.042)	(0.077)		(0.052)	(0.054)
Risk aversion		−0.021	−0.052		0.287^***^	0.289^***^
		(0.054)	(0.043)		(0.083)	(0.093)
Access to electricity		−0.007	−0.020		0.140^***^	0.134^***^
		(0.045)	(0.053)		(0.045)	(0.046)
Access to varietal information		0.035	−0.011		0.119^**^	0.110^*^
		(0.061)	(0.044)		(0.055)	(0.057)
Access to climate information		0.024	0.028		0.134^**^	0.128^**^
		(0.054)	(0.061)		(0.067)	(0.065)
Joint significance of all regressors (F-test)	10.9^***^	5.24^***^	4.8^***^			
79.41^***^						
26.56^***^						
23.45^***^						
*R*^2^	0.04	0.07	0.07	0.21	0.27	0.24
Observations	1,919	1,919	1,919	1,919	1,919	1,919

State-level fixed effects included but not reported. Standard errors clustered at enumeration area level are reported in parentheses, ^***^p < 0.01, ^**^p < 0.05, ^*^p < 0.1. *Note:* Per-capita total expenditure is measured in per capita and transformed in logarithmic scale.

#### (iii) Placebo effects

Herein, we focus on registration without receiving a mobile alert or collecting subsidized fertilizer and improved seeds from redemption centers as a falsification test to examine if our reported results (Table 5, Table 6, Table 7, Table 8) are robust. In particular, our dependent variable takes a value of one if the household is registered for the GES but never received a mobile alert or collected fertilizer and improved seeds and zero otherwise. We used this result as a placebo test because registration without collecting subsidized fertilizer and improved seeds shouldn’t affect productivity and welfare outcome indicators. A positive and significant effect here implies the presence of spurious correlation and hence our reported impacts (from Table 5, Table 6, Table 7, Table 8) cannot be attributed as casual effects of the GES program. We focus on maize yield and income from maize production. We opt to use income from maize production instead of poverty since we based our benefit–cost calculation on GES’s estimated effects on maize income. Results are reported in Table 9. In our placebo test, we did not find any positive and statistically significant effect of the GES on maize yield, maize income, and per-capita total expenditure. This serves as a robustness check for the reported causal impacts of the GES program.

**Table 9:** Placebo effects

	Maize yield	Maize income	Per-capita total expenditure
	With IV	With IV	
GES	−0.384	−0.135	−4.500^***^
	(0.366)	(0.431)	(0.722)
Gender	−0.258^**^	0.079	−0.237
	(0.117)	(0.097)	(0.164)
Land category	0.016	−0.338^***^	−0.143^*^
	(0.066)	(0.056)	(0.082)
GES^*^Gender	0.655	−0.040	−0.270
	(1.102)	(0.881)	(1.411)
GES^*^land category	−0.216	−0.198	0.752
	(0.460)	(0.421)	(0.610)
Education	0.014	0.131^**^	−0.094
	(0.057)	(0.053)	(0.090)
Marital status	−0.034	0.226^***^	−0.204
	(0.106)	(0.069)	(0.157)
Age	0.017	0.001	0.010
	(0.015)	(0.007)	(0.014)
Age squared	−0.000	−0.000	−0.000
	(0.000)	(0.000)	(0.000)
Access to off-farm	0.041	−0.025	0.285^***^
	(0.048)	(0.048)	(0.056)
Roofing material of the house	0.259^***^	0.032	0.156^*^
	(0.068)	(0.062)	(0.082)
Stress shock	0.146^**^	0.204^***^	0.508^***^
	(0.060)	(0.064)	(0.060)
Drought shock	0.040	−0.057	0.055
	(0.069)	(0.044)	(0.081)
Member to credit and saving groups	0.050	0.009	0.210^**^
	(0.060)	(0.078)	(0.097)
Member to labor-sharing groups	−0.136	0.133	0.304^***^
	(0.090)	(0.150)	(0.106)
Member to cooperatives	0.255^***^	−0.000	−0.092
	(0.080)	(0.097)	(0.056)
Member to farmer research group	−0.051	0.037	0.302^***^
	(0.042)	(0.040)	(0.079)
Risk aversion	−0.010	0.133^***^	0.132^***^
	(0.053)	(0.049)	(0.045)
Access to electricity	−0.003	0.030	0.057
	(0.047)	(0.038)	(0.057)
Access to varietal information	0.046	0.144^**^	0.218^***^
	(0.065)	(0.057)	(0.069)
Access to climate information	0.047	0.095^**^	−4.500^***^
	(0.055)	(0.042)	(0.722)
Joint significance of all regressors (F-test)	4.50^***^	8.64^***^	25.92^***^
*R*^2^	0.064	0.10	0.264
Observations	1,919	1,919	1,919

State-level fixed effects included but not reported. Standard errors clustered at enumeration area level are reported in parentheses, ^***^p < 0.01, ^**^p < 0.05, ^*^p < 0.1.

#### (iv) Is the program cost effective?

Herein, we provide benefit–cost ratios albeit without considering indirect benefits and costs. For the purpose of calculating the effectiveness of the program, we collected data on the quantity of seed and fertilizer allocated to farmers as well as the final quantity collected and the price paid by farmers. We calculated the cost of subsidy per kg of fertilizer and improved seed as the difference between the market price and the subsidized price of fertilizer and improved seeds. However, data on registration, administrative, and logistic costs of the GES program are not available despite our best efforts to obtain it. Since costs for registration, transportation, storage, procurement, and administration are likely to be significant; we took some of the cost components of the Malawi input subsidy program from Chirwa and Dorward (2013) to provide a proxy for what the costs of Nigeria’s subsidy program could be.^13^

Table 10 provides benefit–cost ratios of the GES, valued at market prices and without accounting for economy-wide effects. With the gains in maize income as a measure of economic benefits, the benefit–cost ratio of the program is estimated at 1.11. This means that each dollar the government spends on input subsidy generates US$1.11 worth of maize income. Further, the 95% confidence interval suggests that the BCR for all participants of the GES lies between −0.17 and 2.38.

**Table 10:** Cost benefit analysis of the GES

	Benefit	Cost	Benefit–cost ratio	95% CI
Income gain per household	19,727	–		
Total cost per household (subsidy & admin costs)		17,826		
Benefit–cost ratio			1.11	−0.17 to 2.38

It is worth noting, however, that our calculation is based on the so called “production-based” approach and ignores indirect benefits (Arndt, Pauw, & Thurlow, 2015). Using the same production-based approach, Dorward and Chirwa (2011) reported an average benefit–cost ratio of 1.06 for Malawi, whereas Arndt *et al.* (2015) accounted for economy-wide effects and reported a higher benefit–cost ratio of about 1.62 for the same program. Arndt *et al.* (2015) showed that ignoring economy-wide effects can be consequential for large-scale subsidy programs such as the GES. However, these effects stand in contrast to some results from Nigeria, where Takeshima and Liverpool-Tasie (2013) reported that the previous Nigerian input subsidy program has little to no effects on maize prices.

## 5 Conclusions

In Nigeria, improving agricultural productivity through the use of fertilizer and improved seeds is imperative to improve food security and reduce the pervasive nature of rural poverty. However, the use of such key agricultural inputs is rather low even by SSA standard as a result of pervasive input and credit market imperfections. With the premise of improving the use of improved seed and inorganic fertilizer, the government of Nigeria embarked on a mobile phone-based input subsidy program in 2012. Using a unique household-level data from rural Nigeria, this paper examined the productivity and welfare effects of the GES program. In particular, the study provides insights into how effective the program was in enhancing productivity and improving welfare outcomes. The study employed instrumental variable regression approach to control for the potential endogeneity of participation in the GES program.

Our main empirical findings are as follows. First, farmers who participated in the GES increased their maize yield by 26.3%. Similarly, maize income of GES participants increased by ₦19,730. These results suggest that the GES enabled farmers to improve their productivity and income from maize production, which is a case for justifying the intervention. Second, in terms of the welfare outcomes, the GES has a positive and statistically significant effect on per-capita total, food, and non-food consumption expenditures. The size of the estimated effects suggests a large improvement in welfare as a result of participation in the GES program. Specifically, GES participants increased their per-capita total consumption expenditure by 30.7%. As a result of this consumption growth, poverty headcount ratio has declined by 17.7% points among participants of the GES. Third, the effect of the interaction term between participation in the GES and farm land size as well as GES and gender is insignificant suggesting the absence of heterogeneity effects based on gender and farm land size. Fourth, the benefit–cost ratio of the program, valued at market prices and without accounting for economy-wide effects, is about 1.11. Each unit of dollar that the government spends as a subsidy yields a return of 1.11 dollars.

The results presented in this paper should be interpreted with caution. First, our identification strategy relies on cross-sectional data which limits the generalizability of the results beyond one agricultural production year. The use of panel data and fixed-effects would be an important extension in the future. Second, our benefit–cost ratio calculation does not take into account indirect benefits and costs. Moreover, in estimating impacts on productivity and welfare, we did not consider the possibility of farmers re-selling their fertilizer and improved seeds due to lack of data. Although the GES strictly prohibits re-selling of the received inputs, we cannot rule out the possibility of some recipients selling fertilizer and improved seeds in the local market or at the redemption centers. When subsidized inputs are re-sold at the local market, it will definitely introduce a crowding-out effect. Examining the crowding-out effect of the program will therefore be an important extension.

## Figures and Tables

**Table 11:** Validity check for our instrument

Variables	Yield	Maize income	Per-capita total expenditure
Number of years of residence in the village	3.51	180.8	41.7
	(2.2)	(119.6)	(204)
*N*	1,919	1,919	1,919

**Table 12:** Effects on non-food expenditure

	Per-capita non-food expenditure		
	No control	Without IV	With IV
GES	0.61^***^	0.474^***^	0.460^***^
	(0.08)	(0.081)	(0.152)
Gender		−0.166	−0.072
		(0.130)	(0.183)
Land category		−0.156^**^	−0.235^*^
		(0.077)	(0.123)
GES^*^Gender		−0.200	−0.388
		(0.232)	(0.384)
GES^*^land category		0.157	0.317
		(0.132)	(0.223)
Education		0.198^**^	0.235^***^
		(0.091)	(0.091)
Marital status		−0.356^**^	−0.375^**^
		(0.178)	(0.181)
Age		−0.003	−0.003
		(0.014)	(0.016)
Age squared		−0.000	−0.000
		(0.000)	(0.000)
Access to off-farm		0.162^**^	0.136^**^
		(0.066)	(0.069)
Roofing material of the house		−0.082	−0.098
		(0.116)	(0.113)
Stress shock		0.355^***^	0.334^***^
		(0.066)	(0.069)
Member to credit and saving groups		0.068	0.071
		(0.093)	(0.098)
Member to labor-sharing groups		0.019	−0.002
		(0.111)	(0.110)
Member to cooperatives		0.107	0.089
		(0.131)	(0.133)
Member to farmer research group		−0.063	−0.065
		(0.075)	(0.078)
Risk aversion		0.366^***^	0.364^***^
		(0.091)	(0.097)
Access to electricity		0.168^***^	0.174^***^
		(0.060)	(0.061)
Access to varietal information		0.206^***^	0.214^***^
		(0.078)	(0.081)
Access to climate information		0.233^***^	0.222^***^
		(0.074)	(0.083)
Joint significance of all regressors (F-test)	90.75^***^	27.97^***^	26.1^***^
*R*^2^	0.204	0.26	0.245
Observations	1,919	1,919	1,919

State-level fixed effects included but not reported. Standard errors clustered at enumeration area level are reported in parentheses, ^***^p < 0.01, ^**^p < 0.05, ^*^p < 0.1.

## Footnotes

1 To date, many studies have examined the effect of input subsidies in the context of SSA. The most obvious examples include Ricker-Gilbert et al., 2013, Jayne et al., 2013, Holden and Lunduka, 2013, Chirwa and Dorward, 2013 for Malawi. Lunduka et al., 2012, Mason and Ricker-Gilbert, 2013, Mason and Jayne, 2013 for Zambia, Liverpool-Tasie, 2013 for Nigeria. Arguments against subsidies are based on economic inefficiencies and welfare losses as a result of market distortions while arguments for subsidies are largely based on credit and input market imperfections (Holden and Lunduka, 2013, Jayne et al., 2013, Ricker-Gilbert et al., 2013)

2 Usually, a smart subsidy is defined based on the efficiency of targeting. In particular, smart refers to subsidies that work to create demand for commercial fertilizer and seed as opposed to those that have a crowding-out effect. The “smartness” of GES is mostly based on its innovativeness in terms of using mobile-based e-vouchers.

3 However, the absolute magnitude of the subsidy was small given that the government of Nigeria generally allocated less than 3 percent of its budget to agriculture (Takeshima *et al.*, 2013).

4 One may argue that the variable “the number of years the household head has resided in the village” could potentially affect welfare and productivity outcomes. However, our falsification test in the appendix (Table 11), suggests no casual effect of the variable “the number of years the household head has resided in the village” on welfare and productivity outcomes, providing evidence that it is a “plausibly exogenous” IV.

5 Note that, maize income refers to income generated from sales of maize. It could be that some farmers may have lower maize income when harvest is used for own consumption. This is accounted in our food expenditure and total expenditure measures as imputed value of own consumption is included.

6 Official exchange at the time of data collection is (1 USD = 200₦). However, it must be noted that rates are higher than 300₦ in parallel markets.

7 This value is based on World Banks $1.25 per day per capita.

8 Note that all outcome variables except poverty headcount ratio are expressed in logarithmic scale.

9 In principle, collection is a strict subset of receiving a mobile alert. However, some registered farmers didn’t receive alerts due to network coverage issues, loss of mobile phones, and errors while registering mobile numbers. See our robustness checks in Section 4(d).

10 If farmer characteristics—including years-in-village—determine notification (conditional on registration) then it would strongly suggest that receiving a mobile alert is not exogenous to farmers’ action. We thank an anonymous reviewer for pointing this out.

11 Note that we used 3 ha as the median land size in our data is about 3 ha. Moreover, GA (2014) suggested that most eligible farmers are these with a land size of less than 3 ha.

12 Non-food expenditure are presented in the appendix (Table 12).

13 A detail cost structure of the Malawi program is presented by Chirwa and Dorward (2013) on page 122, Table A5.1. Still, not all the cost components of the GES are accounted for due to the lack of cost data from the government. Detail cost components used for the calculation of the BCR are available from authors upon request.
